# Professional image of nursing and midwifery in East Africa: an exploratory analysis

**DOI:** 10.1186/s12912-020-00531-w

**Published:** 2021-03-06

**Authors:** Eunice Wambui Ndirangu, Ahmed Mohammed Sarki, Columba Mbekenga, Grace Edwards

**Affiliations:** 1 School of Nursing and Midwifery, Aga Khan University, Wangiapalla Road, P.O. Box 39349, Nairobi, 00623 Kenya; 2 Nursing Council of Kenya, Kilmani, Kabarnet Rd, Nairobi, Kenya; 3 School of Nursing and Midwifery, Aga Khan University, Colonel Muammar Gaddafi Road, P.O. Box 8842, Kampala, Uganda; 4 Family and Youth Health Initiative (FAYOHI), Dutse, Jigawa State Nigeria; 5 School of Nursing and Midwifery, Aga Khan University, Urambo Street, P.O. Box 38129, Dar es Salaam, Tanzania

**Keywords:** Media image, Nursing, Midwifery, Public perception, Nursing workforce

## Abstract

**Background:**

Evidence suggests that there is a negative image of nursing and midwifery that does not promote these professions as attractive career options. Furthermore, there is a paucity of studies documenting how nursing and midwifery is perceived in East Africa and where such studies exist they are country-specific. The aim of this study was to explore views regarding the image of nursing and midwifery among nurses and midwives in three East African countries, Kenya, Tanzania and Uganda.

**Methods:**

An exploratory descriptive cross-sectional study administered online using Survey Monkey Questionnaires assessed the views and perceptions of nurses and midwives regarding the image of nursing and midwifery professions. Descriptive statistics and Pearson’s Chi square tests were used to analyse the data. The semi-structured questions were analysed using content analysis.

**Results:**

A total of 551 participants took part in the study. The majority were females (61.8%), registered nurses/midwives (45.8%), and aged 30–39 years (34.2%). Most of the respondents were from Kenya (39.7%) and Uganda (32.9%). About two-thirds of the nurses and midwives in this study perceived nursing/midwifery as both trusted and respected professions and expressed having a level of control over how their image was portrayed. Conversely, the nurses and midwives were conscious that the public had mixed responses about the nursing/midwifery professions specifically, some members of the public described nurses/midwives as professionals, knowledgeable and caring, others perceived nurses/midwives to be rude, cruel, unkind, lazy, unkempt, and maids.

**Conclusion:**

This study offers an interesting insight about the image of nursing/midwifery in East Africa. Findings from this study will inform policy makers and educators about key concepts that affect the image of nursing and midwifery in East Africa. The findings will be used to design marketing materials to help improve the image of nursing and midwifery in the region and other African countries.

**Supplementary Information:**

The online version contains supplementary material available at 10.1186/s12912-020-00531-w.

## Background

Globally, the image of nursing and midwifery has been an issue of concern with negative impact for the two professions. Several factors have been associated with the image of nursing and midwifery professions and how it affects patients’ care. Regrettably, factors such as nurse to nurse bullying, hostile clinicians and disruptive behaviour have been found to adversely impact on patient care and reduced work performance [1]. The way patients perceive nurses affects the care relationship, with patients and families who perceive nurses positively also viewing nurses as enhancing their quality of life in long-term care [2]. Hospital work environments may not offer enough autonomy for nurses in their workplaces [3] yet autonomy and decision making affect how nurses and others perceive the power, status and role of nurse midwives in healthcare.

Consistently, the nursing profession has not been adequately represented in the media. A literature review undertaken three decades ago presented four main images of nurses as portrayed by the media, which include the ministering angel, the battleaxe, the naughty nurse and the doctor’s handmaiden [4]. In Sweden, a discourse analysis of text from relevant documents, television programs and various kind of images indicated nursing being portrayed as a female profession where nurses predominantly assumed a subordinate position as assistants to medical doctors [5]. Ultimately, these images influence and shape how the general public and the nurses perceive the nursing profession. Scholars have elucidated a need for disruption of these inappropriate public representations of nursing in media that might be contributing to the shortage of nurses [6–8] where nurses have the central role to play in this.

A study conducted in the Middle East to explore public perceptions of the role of the midwife found that women did not know that such a role existed, despite having received antenatal care at a midwifery led clinic. When questioned about their visibility, the midwives responded that the women referred to them as ‘doctora’ (the physician) and none of the midwives corrected this [9]. In some cases nurses and midwives themselves need to take some responsibility for this, as the image is partly self-created by nurses due to their invisibility and lack of marketing [10]. In another study that explored the image of nursing from nurses’ and non-nurses’ perspective in Pakistan, although 90% of the respondents considered nursing a ‘noble profession’, less than 30% would consider nursing a suitable profession for their own daughters or sisters [11].

In most high-income countries, the public understands and appreciates the role of nurses and midwives. In addition, nurses and midwives consistently top public surveys about professionals that are trusted, yet there is a shortage of suitable applicants for both professions [12, 13]. However, the public admiration for nurses and midwives in these countries does not appear to encourage or motivate young people to choose nursing and midwifery as a career choice [14].

In low- and middle-income countries, the shortage of nurses and midwives is marked [15] and there are varying views regarding nurses and midwives. In South Africa, the results of a study on the image of nurses and nursing as perceived by the South African public showed that despite the negative image portrayed by the media, there is a generally positive public response to nursing and nurses in South Africa. However, only 43% of the respondents indicated that they would want their children to become nurses. Nursing was not viewed as a career choice and the public did not seem to understand the complexities of a nursing career [16]. In Nigeria, nurses working in tertiary hospitals negatively perceived their profession influencing their motivation to continue practice within the profession [17]. In India, 25% of respondents in an image of nursing and career influence study stated that the public perceive nursing as a dirty job and nurses as being rude to patients [18].

In East Africa, the shortage of nurses compared to international averages is clear. According to the World Health Organization [19], East Africa has approximately 0.94 nurses and midwives per 1000 inhabitants. This falls below the African region average of 1.2 per 1000 and the global average of 2.92 nurses per 1000. The State of the World’s Midwifery Report in 2014, highlights that the unmet needs for nurses and midwives is up to 55% in Kenya, 26% in Tanzania and 73% in Uganda [20]. This shortage of nurses and midwives may be due, in part, to the image and perception the general public and other professionals have on nursing and midwifery professions. However, published evidence suggests mixed perceptions about the nursing and midwifery professions. For instance, in Tanzania, a study that assessed knowledge and attitudes of secondary school students towards the nursing profession report that students do not opt to join nursing programs due to the image they have of the profession from the public [21]. On the contrary, a study in Kenya shows that majority of the first year nursing students were positive about the profession and 90% would still choose nursing as a career if given a second chance [22].

In order to explore contributors to the shortage of nurses and midwives, the views of nurses, midwives, medical doctors and other health care professions were sought to elicit perceptions of the role and image of nursing and midwifery in three East African countries, Kenya, Tanzania and Uganda. However, due to limited numbers, data from medical doctors and other health care professions were excluded in the analysis, and therefore this paper present results from nurses and midwives only.

### Study rationale

The majority of existing studies reviewed about image of nursing and midwifery were conducted in the context of high income countries in Europe and North America. Although these studies have established that members of their populations held higher regard for nurses than other professionals [23] other countries professionals ranked nurses and midwives lower than doctors [24]. Furthermore, nurses regarded their role in a more positive image than their patients and fellow health care workers such as doctors [24].

In addition, there is a paucity of studies documenting how nursing is perceived in East Africa. Therefore, it is of interest to investigate the nurses and midwives’ perception regarding the image of nursing and midwifery in the East African context. This study is of ultimate importance to better understanding and improving the image of nursing and midwifery within East Africa. It attempts to fill a knowledge gap arising from lack of similar research on the topic within the region. In addition, it aims to generate scientific evidence for supporting nursing and midwifery associations to positively promote nursing and midwifery and address negative perceptions of the profession.

### Objectives

1. To determine views of nurses and midwives regarding the image of nursing and midwifery across East Africa
2. Identify strategies for promoting a positive image of nursing and midwifery in East Africa

## Methods

### Study design

This was an exploratory cross-sectional descriptive study with use of online quantitative survey tools consisting of structured, semi-structured questions and Likert scales. A questionnaire was designed to assess the view and perceptions of nurses and midwives regarding the image of nursing and midwifery professions.

### Study setting

This study was conducted across three East African countries namely Kenya, Tanzania, and Uganda.

### Study population

All registered nurses and midwives who are members of nursing and midwifery professional associations or regulatory councils in the study settings.

### Inclusion criteria

Respondents were recruited into the study if they meet the following criteria:

I. Nurses and midwives in active practice within East African health care sector.
II. Working in East Africa public or private sector.
III. Belonging to or being a member professional association or regulatory council within the country.

### Exclusion Criteria

I. Expatriate nurses and midwives working within the three East African countries.

### Data collection tool

Data was collected using a questionnaire developed for this study (see Supplementary file 1) led by GE with input from EWN and CM- together they have over 50 years of practicing nursing and midwifery between them. The questionnaire was piloted with a convenient sample of nurses and midwives and corrections were made to ensure that the questions were well defined and clearly understood [25]. The questionnaire focused on the nurses and midwives’ perception of their own profession. In addition, doctors’ perception of the profession of nursing and midwifery was sought (although due to the paucity of responses from the doctors, their data was not included in the analysis). The questionnaire comprised structured- and semi-structured questions focusing on three main areas, namely: 1) view of nurses and midwives regarding their own profession; 2) perception of how other healthcare professionals and members of the public view nursing and midwifery profession and their role; and, 3) how the image and perceptions of nursing and midwifery can be improved.

### Data collection procedures

We administered questionnaires to the nurses/midwives through Survey Monkey- an online platform for administering questions to respondents who may not be reached for face-to-face interviews. After uploading the questionnaire on the Survey Monkey platform, a unique link was sent to each participant. The invitation was a single use invitation link that was deactivated once a respondent completed the questionnaire, to prevent study respondents from repeating participation in the study. The keyed in responses was automatically be encrypted and collated in the Survey Monkey database.

### Sample size

The sample size was calculated using the Thrusfield formula [26] for estimating proportions defined as:

$$ n=(zd)2 pq. $$

Where: n = Sample size.

z = Linked to 95% confidence interval.

p = Expected proportion of nurses and midwives imaging.

q = 1-p.

d = Relative desired precision.

Given that we are not aware of the value of the expected proportion, *p* the study used 50%, assuming 95% level of significance and 5% desired precision.

The estimated number for the study was 385. However, due to finite populations of nurses and doctors of approximately 40,000 in the three countries, a total of 382 nurses, midwives, and doctors were required for the study after adjusting based on the following formula:

n (adj) = (Nxn)/(N+n) Of note, due to the limited number of responses from the doctors, the data from the doctors were excluded in the analysis.

### Sampling technique

The research team worked with the leadership of the professional associations to randomly selected participants from the list of members using MS Excel. The selected participants were then invited via email by the Secretaries of the professional associations containing information on the study, consent form and a personalized link to the tool. Table 1 shows the definition of some of the variables.

**Table 1:** Definition of Variables

Term	Definition
Age	In years
Gender	Male/Female/Others
Profession	Nursing/midwifery
License category	Registered Nurse (RN), Enrolled Nurse (EN)
Experience	In years
Image	Image is a summary of how nursing and midwifery profession is perceived by nurse/midwives and other disciplines. It was measured using an image index generated using principal component analysis of the following:
	• Professional View of Nurses/midwives
	• Impact of Nurses/midwives on others
	• Other healthcare professionals’ perceptions of Nursing & Midwifery Professions.
	• Nurses and Midwives belief of how the public perceive their professions.

### Data management

All data were kept confidential and stored securely in accordance to research ethics governing human participants. No identifying information was stored, and only basic demographic data was collected. Data was stored within AKU secured electronic folders and only accessible by the investigators of the study.

### Data analysis

Data was checked for completeness and consistency and then entered into SPSS version 24 for analysis. Descriptive statistics was used to describe; sample characteristics, professional views of the nurses, impact of nurses on others, and belief of how others perceive their profession. However, owing to limited data, information provided by doctors and other professionals were excluded from the analysis. There were questions with incomplete responses that were missing at random. Missing data due to non-response from the study participants was managed using listwise deletion [27, 28]. However, given the relative small sample of our participants, the data was analyzed as it was and we are confident that the missing data did not undermine the findings of this study.

Comparison between how nurses/midwives perceive their profession versus how other professionals perceive the nursing profession was analyzed using Pearson’s chi-square test [29]. Other comparisons include between categories of license (RN/EN) between country (Kenya, Tanzania & Uganda) using chi-square test with a *p*-value of less than 0.05 considered statistically significant. In addition, the semi-structured questions in our questionnaire (see questions 5 & 6 supplementary file 1) were analyzed using content analysis [30] specifically to reveal common patterns in the data. The content analysis was carried out manually, three authors EWN GE, and CM conducted the analysis while AMS double-checked the findings.

## Results

A total of 551 participants took part in the study (Table 2), however, we only included data for nurses and midwives (511 participants) in the analysis. Majority were females (*n*=316, 61.8%), aged 30–39 years (*n*=172, 34.2%) and registered nurses/midwives (*n*=234, 45.8%), followed by registered community health nurses (*n*=121, 23.7%), and enrolled nurse/midwife (*n*=48, 9.4%). A majority of the respondents were from Kenya (*n*=203, 39.7%) and Uganda (*n*=168, 32.9%). Among the respondents from Kenya, the mean age was 38.5 years (SD: 9.4 years) and average years of practice was 14.4 (9.7) years.

**Table 2:** Characteristics of the study participants by Country

	Kenya (n, %)	Uganda (n, %)	Tanzania (n, %)	Total (n, %)	***p***-value
**Nationality / Sample**	203 (39.7)	168 (32.9)	140 (27.4)	511	
**Age, years**					
**Age (Mean, SD)**	38.5 (9.4)	39.4 (13.1)	37.5 (10.6)		
20–29	38 (19.1)	47 (28.3)	41 (29.2)	126 (25.0)	
30–39	73 (36.7)	54 (32.5)	45 (32.1)	172 (34.2)	0.099
40–49	55 (27.6)	30 (18.1)	27 (19.2)	112 (22.3)	
50+	33 (16.6)	35 (21.1)	25 (17.9)	93 (18.5)	
**Gender**					
Male	79 (38.9)	46 (27.4)	70 (50.0)	195 (38.2)	0.000
Female	124 (61.1)	122 (72.6)	70 (50.0)	316 (61.8)	
**Professional Groups**					
Enrolled nurse/midwife	1 (0.5)	31 (18.5)	16 (11.4)	48 (9.4)	
Registered nurse/midwife	40 (19.7)	92 (54.8)	102 (72.9)	234 (45.8)	0.000
Registered Community Health nurse	116 (57.1)	5 (3.0)	0 (0.0)	121 (23.7)	
**Highest Qualification**					
Certificate	1 (0.5)	34 (20.2)	15 (10.7)	50 (9.7)	
Diploma	105 (51.7)	58 (34.5)	45 (32.1)	208 (40.7)	0.000
Degree	72 (35.5)	50 (29.8)	47 (33.6)	169 (33.1)	
Master/PhD	25 (12.3)	26 (15.5)	33 (23.6)	84 (16.4)	
**Years of Practice**					
Mean (SD)	14.4 (9.7)	15.6 (12.8)	12.1 (10.3)		
0–5	43 (21.6)	40 (24.2)	47 (35.1)	47 (35.1)	
6–10	44 (22.1)	37 (22.4)	33 (24.6)	33 (24.6)	
11–15	33 (16.6)	22 (13.3)	16 (11.9)	16 (11.9)	0.084
16–20	30 (15.1)	23 (13.9)	8 (6.0)	8 (6.0)	
20+	49 (24.6)	43 (26.1)	30 (22.4)	30 (22.4)	
**Direct patient care**					
Yes	172 (84.7)	141 (83.9)	108 (77.1)	421 (82.4)	0.158
No	31 (15.3)	27 (16.1)	32 (22.9)	90 (17.6)	
**Does public perception of nursing and/or midwifery determine how nurses and/or midwives conduct their work**					
Yes	89 (55.3)	68 (44.4)	37 (36.6)	194 (46.7)	
No	72 (44.7)	85 (55.6)	64 (63.4)	221 (53.3)	0.010

### Nurses/midwives professional view

In Table 3, most nurses/midwives strongly agree or agree that nursing and midwifery is a calling (87.2%), and that they are powerful (87.5%) and trusted professions (82.5%). Nurses also strongly agreed that they have a level of control over how their image was portrayed (84.9%) and that they are effective advocates for their profession (71.1%).

**Table 3:** Nurses and midwives professional view

	Strongly disagree/ Disagree (n, %)	Not Sure (n, %)	Strongly Agree/ Agree (n, %)
Nursing and/or midwifery is my calling	43 (8.5)	22 (4.4)	441 (87.2)
My family wanted me to become a nurse and/or midwife	187 (37.0)	92 (18.2)	226 (44.8)
There is a shortage of nurses and/or midwives in my country	81 (16.0)	13 (2.6)	411 (81.4)
Nursing and/or midwifery are powerful professions	54 (10.7)	9 (1.8)	441 (87.5)
Nursing and/or midwifery are trusted professions	50 (10.0)	38 (7.6)	414 (82.5)
Nursing and/or midwifery are respected professions	165 (32.7)	46 (9.1)	294 (58.2)
Nurses and/or midwives can control how their image is portrayed	42 (8.3)	34 (6.8)	428 (84.9)
Nurses and/or midwives are affectively assertive	103 (20.6)	80 (16.0)	318 (63.5)
Nurses and/or midwives are effective advocates for their profession	115 (22.9)	30 (6.0)	356 (71.1)
Nurses and midwives have pride in their profession	109 (21.7)	63 (12.6)	330 (65.7)

### Nurses/midwives impact on others

As shown in Table 4, female nurses generally felt that how nurses present themselves to their patients mattered a lot (84.3%). Both male and female nurses felt that how nurses greet their patients had a great effect on the image of nurses/midwives (77.3% vs 76.5%). On the other hand, female nurses pointed out that having updated skills and knowledge on practice among nurses had a big effect on their image (80.1%).

**Table 4:** Nurses/Midwives Impact on Others by gender

	No effect (n, %)			Little effect (n, %)			Great effect (n, %)		
	Male	Female	Total	Male	Female	Total	Male	Female	Total
How nurses and/or midwives present themselves to patients	2 (1.1)	9 (2.9)	11 (2.2)	39 (20.6)	40 (12.8)	79 (15.8)	148 (78.3)	263 (84.3)	411 (82.0)
Whether patients and families feel that nurses and/or midwives show compassion	5 (2.7)	12 (3.9)	17 (3.4)	61 (32.6)	123 (39.6)	184 (37.0)	121 (64.7)	176 (56.6)	297 (59.6)
How nurses and/or midwives dress and appear at work	8 (4.3)	17 (5.5)	25 (5.0)	39 (20.7)	52 (16.9)	91 (18.4)	141 (75.0)	239 (77.6)	380 (76.7)
How nurses and/or midwives are portrayed in the media	16 (8.5)	17 (5.5)	33 (6.7)	58 (30.9)	76 (24.8)	134 (27.1)	114 (60.6)	214 (69.7)	328 (66.3)
How nurse and/or midwives greet patients	7 (3.8)	16 (5.1)	23 (4.6)	35 (18.9)	57 (18.3)	92 (18.6)	143 (77.3)	238 (76.5)	381 (76.8)
Whether nurses and/or midwives keep their skills and knowledge up to date	7 (3.8)	10 (3.2)	17 (3.4)	48 (25.8)	52 (16.7)	100 (20.1)	131 (70.4)	250 (80.1)	381 (76.5)
How nurses and/or midwives behave professionally	3 (1.6)	9 (2.9)	12 (2.4)	32 (17.3)	47 (15.1)	79 (15.9)	150 (81.1)	256 (82.1)	406 (81.7)
How medical professionals interact with nurses and/or midwives	10 (5.5)	12 (3.9)	22 (4.5)	50 (27.3)	88 (28.5)	138 (28.1)	123 (67.2)	209 (67.6)	332 (67.5)

Moreover, as highlighted in Table 5, nurses in Kenya felt that factors such as how nurses/midwives greet patients (84.3%), nurses keeping their skills and knowledge up to date (85%) and nurses behaving professionally (93%) have a great effect on their image. Nurses in Uganda felt that how medical professionals interact with nurses/midwives had a great effect (74.9%) on their image.

**Table 5:** Nurses/Midwives Impact on others by nationality

	No effect (n, %)			Little effect (n, %)			Great effect (n, %)		
	Kenya	Uganda	Tanzania	Kenya	Uganda	Tanzania	Kenya	Uganda	Tanzania
How nurses and/or midwives present themselves to patients	0 (0.00)	1 (0.6)	10 (7.4)	16 (8.0)	18 (10.8)	45 (33.3)	183 (92.0)	148 (88.6)	80 (59.3)
Whether patients and families feel that nurses and/or midwives show compassion	7 (3.5)	4 (2.4)	6 (4.6)	54 (27.1)	61 (36.3)	69 (52.7)	138 (69.4)	103 (61.3)	56 (42.8)
How nurses and/or midwives dress and appear at work	6 (3.0)	5 (3.0)	14 (10.5)	28 (14.1)	27 (16.4)	36 (27.1)	164 (82.8)	133 (80.6)	83 (62.4)
How nurses and/or midwives are portrayed in the media	8 (4.0)	39 (23.8)	51 (38.6)	44 (22.1)	39 (23.8)	51 (38.6)	147 (73.9)	115 (70.1)	66 (50.0)
How nurse and/or midwives greet patients	4 (2.0)	7 (4.2)	12 (9.2)	27 (13.6)	23 (13.8)	42 (32.1)	167 (84.3)	137 (82.0)	77 (58.8)
Whether nurses and/or midwives keep their skills and knowledge up to date	3 (1.5)	5 (3.0)	9 (6.8)	27 (13.5)	29 (17.5)	44 (33.3)	170 (85.0)	132 (79.5)	79 (59.9)
How nurses and/or midwives behave professionally	0 (0.00)	2 (1.2)	10 (7.6)	14 (7.0)	25 (15.1)	40 (30.3)	185 (93.0)	139 (83.7)	82 (62.1)
How medical professionals interact with nurses and/or midwives	5 (2.5)	7 (4.3)	10 (7.6)	50 (25.3)	34 (20.9)	54 (41.2)	143 (72.2)	122 (74.9)	67 (51.2)

### Image and perception of nursing and midwifery by other health care professionals

Generally, nurses in the three study areas (Kenya, Tanzania, and Uganda) felt strongly that they were respected and valued (61.7%). More than half (58.2%) of the nurses in Uganda strongly disagreed that medical doctors have a high regard for them as nurses (Table 6).

**Table 6:** Health care professionals’ perception of nursing and midwifery by gender as reported by nurses/midwives

Doctors’ and other health care professionals’ image and perception of nursing and midwifery	Strongly disagree / Disagree (n, %)		Not Sure (n, %)		Strongly Agree/ Agree (n, %)	
	Male	Female	Male	Female	Male	Female
I feel respected and valued in my work as a nurse and/or midwife	65 (35.1)	87 (28.2)	17 (9.2)	20 (6.5)	103v (55.7)	202 (65.4)
Medical doctors have a high regard for nurses and/or midwives	104 (56.2)	140 (45.3)	17 (9.2)	31 (10.0)	64 (34.6)	138 (44.7)
Other health professionals have a high regard for nurses and/or midwives	71 (39.0)	117 (38.0)	32 (17.6)	48 (15.6)	79 (43.4)	143 (46.4)
Medical doctors regard nurses and/or midwives as equal partners in patient care	116 (63.7)	183 (59.2)	15 (8.2)	25 (8.1)	51 (28.0)	101 (32.7)
Advancing my nursing/midwifery knowledge and competencies improves my image as a nurse and/or midwife	11 (6.0)	15 (4.9)	1 (0.6)	4 (1.3)	170 (93.4)	289 (93.8)
Nurses and/or midwives have autonomy in making decisions regarding patient care	80 (44.0)	115 (37.6)	12 (6.6)	18 (5.9)	90 (49.5)	173 (56.5)
Collaboration between physicians and nurses and/or midwives improves patient health outcomes	6 (3.3)	10 (3.3)	1 (0.6)	0 (0.00)	175 (96.2)	298 (96.8)

#### Perceived roles of nurses and/or midwives

### Nurses/midwives perception of their role

Nurses/midwives perceive their role as that of advocating for the patient, taking care of patients, promoting health, nursing and supervisory as well as policy making (Table 7).

**Table 7:** Public image by nationality as reported by nurses/midwives

	Strongly disagree / Disagree (n, %)			Not Sure (n, %)			Strongly Agree/ Agree (n, %)		
	Kenya	Uganda	Tanzania	Kenya	Uganda	Tanzania	Kenya	Uganda	Tanzania
The public has a high regard for nurses and/or midwives	70 (36.3)	44 (26.8)	39 (31.7)	25 (13.0)	6 (3.7)	27 (22.0)	98 (50.8)	114 (69.5)	57 (46.3)
Nurses and/or midwives are not valued by society	108 (56.3)	103 (62.8)	34 (27.9)	14 (7.3)	10 (6.1)	24 (19.7)	70 (36.5)	51 (31.1)	64 (52.5)
My professional behaviour affects my image	13 (6.8)	24 (14.6)	35 (29.4)	5 (2.6)	4 (2.4)	18 (15.1)	174 (90.6)	136 (82.9)	66 (55.5)
Showing compassion and care is important	2 (1.1)	1 (0.6)	4 (3.3)	0 (0.0)	0 (0.0)	1 (0.8)	189 (99.0)	163 (99.4)	117 (95.9)
It is important for nurses and/or midwives to have good customer service skills	5 (2.6)	1 (0.6)	3 (2.5)	3 (1.6)	1 (0.6)	1 (0.8)	183 (95.8)	161 (98.8)	117 (96.7)
Marketing nursing and/or midwifery to the public is important	9 (4.8)	12 (7.3)	4 (3.3)	5 (2.7)	6 (3.7)	6 (5.0)	174 (92.6)	146 (89.0)	110 (91.7)
Humanity is an essential component of my role	5 (2.6)	0 (0.0)	4 (3.3)	0 (0.0)	1 (0.6)	1 (0.8)	186 (97.4)	163 (99.4)	116 (95.9)
The public has a positive image of nursing and/or midwifery	70 (37.0)	56 (34.2)	45 (37.5)	30 (15.9)	17 (10.4)	32 (26.7)	89 (47.1)	91 (55.5)	43 (35.8)
Nursing and/or midwifery have varied career paths	34 (17.8)	14 (8.6)	15 (12.4)	8 (4.2)	6 (3.7)	20 (16.5)	149 (78.0)	143 (87.7)	86 (71.1)
Nursing and/or midwifery is challenging and rewarding	14 (7.5)	11 (6.7)	13 (10.7)	7 (3.7)	4 (2.4)	7 (5.8)	167 (88.8)	149 (90.9)	101 (83.5)

### Perception of the role of nurses and/or midwives by other health care professionals as reported by nurses/midwives

Most nurses/midwives reported that other healthcare professionals perceive the role of nurses as being professional, offering care in an assertive and compassionate manner. They perceive nurses to be provider of patient care and timely communication. They believe nurses are knowledgeable, competent, experienced, and respected.

### How do members of the public perceive the role of nurses and/or midwives?

The public perceive the role of the nurses as that of providing care to patients in a compassionate, caring, kind, respectful and professional manner. Specifically, nurses are perceived to have good knowledge, customer care skills, approachable, kind, and competent and have effective communication skills (Table 8).

**Table 8:** Public image by gender

	Strongly disagree/ Disagree (n, %)		Not Sure (n, %)		Strongly Agree/ Agree (n, %)	
	Male	Female	Male	Female	Male	Female
The public has a high regard for nurses and/or midwives	52 (29.6)	101 (33.2)	21 (11.9)	37 (12.2)	103 (58.5)	166 (54.6)
Nurses and/or midwives are not valued by society	83 (47.4)	162 (53.5)	22 (12.6)	26 (8.6)	70 (40.0)	115 (38.0)
My professional behaviour affects my image	22 (12.6)	50 (16.7)	8 (4.6)	19 (6.3)	145 (82.9)	231 (77.0)
Showing compassion and care is important	3 (1.7)	4 (1.3)	0 (0.0)	1 (0.3)	172 (98.3)	297 (98.3)
It is important for nurses and/or midwives to have good customer service skills	3 (1.7)	6 (2.0)	3 (1.7)	2 (0.7)	167 (96.5)	294 (97.4)
Marketing nursing and/or midwifery to the public is important	9 (5.2)	16 (5.4)	2 (1.2)	15 (5.0)	162 (93.6)	268 (89.6)
Humanity is an essential component of my role	2 (1.2)	7 (2.3)	2 (1.2)	0 (0.0)	170 (97.7)	295 (97.7)
The public has a positive image of nursing and/or midwifery	60 (34.5)	111 (37.1)	29 (16.7)	50 (16.7)	85 (48.9)	138 (46.2)
Nursing and/or midwifery have varied career paths	22 (12.6)	41 (13.6)	11 (6.3)	23 (7.6)	141 (81.0)	237 (78.7)
Nursing and/or midwifery is challenging and rewarding	16 (9.3)	22 (7.3)	8 (4.7)	10 (3.3)	148 (86.1)	269 (89.4)

#### Attitudes towards nurses and midwives

The attitude toward nurses and midwives was explored using semi-structured questions from our questionnaire. The following sub-headings present the findings.

### Attitude of healthcare workers towards nurses and/or midwives

The nurses/midwives surveyed believe that other healthcare workers are perceived to have varying attitudes about nurses/midwives. While some healthcare professionals view nurses/midwives as professional, knowledgeable, and caring. Some healthcare workers view nurses/midwives as inferior, rude, lazy, lacking, less, dirty and maids.

### Public attitude of members of the public towards nurses and/or midwives

Similar to the perception of other healthcare workers, the members of the public also have both positive and negative views about nurses and midwives. The public views them as trustworthy, educated, respected, savers and helpers. However, they also view them as doctors, rude, thieves, failures, less, cruel, unkind, corrupted, harsh, poor and maids.

## Discussion

To our knowledge, this is the first multi-site study undertaken to explore the professional image of nursing and midwifery in East Africa using the lens of nurses and midwives. Of note, the concept of image has been a longstanding challenge to the nursing profession. Our key findings present two distinct perspectives. On one hand, the nurses/midwives appeared to have a more positive viewpoint about their professions. For example, about two-thirds of the nurses and midwives perceived nursing/midwifery as both trusted and respected professions. In addition, they expressed having a level of control over how their image was portrayed and that they are effective advocates for their profession. They also strongly feel valued and respected by doctors, other healthcare professionals, and members of the public.

From the perspective of the respondents, healthcare workers and the public had mixed responses about nurses/midwives. For example, healthcare workers think that nurses/midwives are professionals, knowledgeable and caring. Similarly, the respondents indicated that some members of the public perceive nurses/midwives as trustworthy, savers, helpers and also knowledgeable. However, other doctors, healthcare workers and members of the public had negative perceptions about nurses/midwives. Specifically, some nurses/midwives are perceived by both groups to be rude, cruel, unkind, lazy, unkempt, and maids.

The diverse findings reported in our study are comparable with previous studies. For example, Takase, Maude, and Manias [31] conducted a mixed-methods study comparing nurses’ self-image versus their perceived public image in Australia and how the latter affects their work behaviour, the authors reported that nurses rated their aptitude for leadership more positively than they thought the public viewed them. In addition, Takase and colleagues [31] found that both self-image and public-image were significant in predicting job behaviours. Similarly, several studies show that nurses have positive perception about their profession, a comparative study conducted among Egyptian and Jordanian male nursing students [32] reported that the participants in both countries were highly positive and optimistic about their profession independent of the views of the public, albeit the latter was considered crucial by the male nurses. Additionally, a qualitative study conducted in Tanzania, East Africa, showed that nurses feel professional pride which makes them discharge their duties independent of public perceptions, though the nurses expressed some limitations and inadequacies- related to staff shortages and inadequate equipment to provide optimal care [33].

The positive perceptions and self-image described by the nurses/midwives in our study could potentially be explained by other factors such as the limited interaction of nurses/midwives with the public. For example, in a discussion paper, [10] explored the public image versus self-concept and professional identity of nurses and similar to our results, ten Hoeve and colleagues reported that the public image of nursing is diverse and incongruent. The authors also posited that nurses’ invisibility was a major contributor of the incongruity and diverse views of the profession in the public domain, thus, recommending that nurses utilize all available media including social media and the internet to promote their profession and increase their visibility.

In contrast, studies conducted in Germany revealed that nursing as a profession was perceived positively by the public than the nurses themselves [34], owing to lower job satisfaction and low self-esteem among the nurses. It is not uncommon for nurses to have a negative perception about their profession, for example, a cross-sectional survey that examined the professional image of nursing among nurses working in tertiary hospitals in Southeastern Nigeria [17] revealed that majority of the participants had negative perceptions about the profession, citing factors such as poor remuneration, stereotyping of the profession, and low levels of appreciation among others.

Furthermore, a recent systematic review on contemporary public perceptions of nursing [35] found the following factors to guide public perception of nurses: misrepresentation of the nursing profession by the media; incongruence- nurses not respected but trusted; poor understanding of the role of nurses; and how entertainment value was derived from demeaning nurses. Albeit not explicit, the negative perception of nurses/midwives reported in our study echo the findings of the review by Girvin and colleagues [35], such that even among healthcare workers, there seems to be limited appreciation and understanding of the core role of nursing/midwifery. Extraordinarily, as shown in our study and previous studies [10, 34, 36], the appearance and behaviour of a few nurses/midwives is often used to characterize and define the image of nursing/midwifery- the largest healthcare profession in the world [37].

It is imperative to acknowledge that nurses and midwives will be an integral part of achieving universal health coverage globally. However, with the ever-increasing challenges such as chronic staffing shortages, inadequate equipment, uneven distribution of nurses/midwives, low remunerations, and increased pressure from changing disease patterns facing the nursing profession in low- and middle-income countries, the need to strengthen nursing and midwifery cannot be overemphasized. Specifically, the pervasive healthcare staffing shortage in many East African countries leads to task shifting in many of these countries where nurses have been reportedly carrying the burden of care for other professions [38, 39]. It is not uncommon to find nurses in East Africa taking up the role of doctors, lab technicians, pharmacists etc. exposing them to working beyond their scope of practice without any training or relevant skills and legal protection. This task shifting may influence the image of nursing/ midwifery with respect to how they perceive themselves and their profession and how others perceive them.

In the present study, the three countries (Kenya, Tanzania, and Uganda), require a multi-stakeholder approach to address the factors responsible for the negative perceptions of nurses/midwives by the public and healthcare workers. These approaches should include long-term innovative strategies that include the use of social media platforms and increased public engagement to promote nursing and midwifery. Remarkably, over one-third of our participants were males- indicating that nursing and midwifery are embraced as career options by the male gender in the East African region. These figures are promising particularly because nursing as a profession suffers from public stereotyping and historically perceived to be a female-dominated profession [40].

In spite the strengths of our study, a few limitations exist. This initial study used a quantitative approach hence in depth exploration of some the findings was not possible. Further, while the initial intent was to examine the image of the nurse from the perception of nurses, midwives and doctors, the response rate from the doctors was very low making it difficult to do any meaningful statistical analysis. Thus, there were incomplete results in the findings. Although we acknowledge that triangulating data collection methods and sources of data is beneficial, this may come in a regional follow up study that will include doctors and other health workers that work directly with nurses and midwives. Another limitation is that the public’s perception was explored through the viewpoint of nurses and midwives and not directly from the public. Much as this could be a limitation, it highlights that the nurses and midwives in the three countries are acutely aware of how they and their profession are perceived in the public. In addition, there was incomplete responses to some questions and we decided to analyze the data as is but we are confident that the missing data in this study did not undermine the findings.

In view of our findings, we propose some recommendations. First, we recommend that the Nursing and Midwifery Councils and Associations of Kenya, Tanzania and Uganda employ strategies such as on-going context-specific continuing professional development trainings (CPDs) geared towards enhancing nurse and midwife self-image. Second, the Councils and Associations need to develop and improve existing guidelines on ‘how nurses and midwives’ interact with patients. Third, there is need for a detailed examination of public’s perception of nurses and midwives in the three countries and the East Africa region.

## Conclusion

In conclusion, our study offers an interesting insight about the image of nursing/midwifery in East Africa and we recognize that more work needs to be done in the region to promote this noble profession. Findings from this study will inform policy makers and educators about key concepts that affect the image of nursing and midwifery in East Africa. The findings will be used to inform further research and design marketing materials to help improve the image of nursing and midwifery in the region and other African countries.

## Supplementary Information

**Additional file 1.** Study questionnaire.

## Data Availability

The data generated or analyzed in this study are available from the corresponding author on reasonable request.
