# Supplementary material for: Professional image of nursing and midwifery in East Africa: an exploratory analysis
Source: BMC Nurs. 2021 Mar 6;20:37. doi: 10.1186/s12912-020-00531-w (PMC7936462; doi:10.1186/s12912-020-00531-w)
Supplement: Supplementary file 1 — Additional file 1. Study questionnaire. [file 12912_2020_531_MOESM1_ESM.doc]

**Introduction**

**Perceptions of Nursing and Midwifery in East Africa Study**

Thank you for responding to this link. We are conducting a study to explore the views of nurses, midwives and physicians regarding the perceptions of health care professionals on the image of nurses and midwives. Participation is voluntary, and you are free to withdraw from this project at any time. Your responses are completely confidential, and your anonymity is assured. The results will be shared using aggregated data.

**If you are happy to continue please tick here**

I declare that I have read and understood the aim of this research and the nature of participation. I hereby give agree to participate in the perceptions of nursing and midwifery survey.

**Nurses/ Midwives Questionnaire**

**Nationality (please select one)**

Kenyan 🞏

Ugandan 🞏

Tanzanian 🞏

Other (Please state) 🞏

**1. Your Professional Views**

*Please tick the box you feel is correct*

|  | **Strongly disagree** | **Disagree** | **Not Sure** | **Agree** | **Strongly**  **Agree** |
| --- | --- | --- | --- | --- | --- |
| Nursing and/or midwifery is my calling |  |  |  |  |  |
| My family wanted me to become a nurse and/or  midwife |  |  |  |  |  |
| There is a shortage of nurses and/or midwives in my  country |  |  |  |  |  |
| Nursing and/or midwifery are powerful professions |  |  |  |  |  |
| Nursing and/or midwifery are trusted professions |  |  |  |  |  |
| Nursing and/or midwifery are respected professions |  |  |  |  |  |
| Nurses and/or midwives can control how their image is  portrayed |  |  |  |  |  |
| Nurses and/or midwives are affectively assertive |  |  |  |  |  |
| Nurses and/or midwives are effective advocates for their  profession |  |  |  |  |  |
| Nurses and midwives have pride in their profession |  |  |  |  |  |

**2. Your Impact on Others**

**2.1 What effect do you think the following statements have on the image of nurses/midwives?**

*Please tick the box you feel is correct*

|  | No  effect | Little  effect | Great  effect |
| --- | --- | --- | --- |
| How nurses and/or midwives present themselves to patients |  |  |  |
| Whether patients and families feel that nurses and/or midwives show  compassion |  |  |  |
| How nurses and/or midwives dress and appear at work |  |  |  |
| How nurses and/or midwives are portrayed in the media |  |  |  |
| How nurse and/or midwives greet patients |  |  |  |
| Whether nurses and/or midwives keep their skills and knowledge up to date |  |  |  |
| How nurses and/or midwives behave professionally |  |  |  |
| How medical professionals interact with nurses and/or midwives |  |  |  |

3. **Doctors’ and other health care professionals image and perception of nursing and midwifery**

*Please tick the box you feel is correct*

|  | **Strongly disagree** | **Disagree** | **Not**  **Sure** | **Agree** | **Strongly**  **Agree** |
| --- | --- | --- | --- | --- | --- |
| I feel respected and valued in my work as a nurse and/or  midwife |  |  |  |  |  |
| Medical doctors have a high regard for nurses and/or  midwives |  |  |  |  |  |
| Other health professionals have a high regard for nurses  and/or midwives |  |  |  |  |  |
| Medical doctors regard nurses and/or midwives as equal  partners in patient care |  |  |  |  |  |
| Advancing my nursing/midwifery knowledge and  competencies improves my image as a nurse and/or midwife |  |  |  |  |  |
| Nurses and/or midwives have autonomy in making decisions  regarding patient care |  |  |  |  |  |
| Collaboration between physicians and nurses and/or  midwives improves patient health outcomes |  |  |  |  |  |

3.1 For doctors who value and appreciate the role of nurses and/or midwives, what is the aspect they value most? *(List top three attributes)*

**4. Public image and perception of nursing and midwifery**

*Please tick the box you feel is correct*

|  | **Strongly**  **disagree** | **Disagree** | **Not**  **Sure** | **Agree** | **Strongly**  **Agree** |
| --- | --- | --- | --- | --- | --- |
| The public has a high regard for nurses and/or midwives |  |  |  |  |  |
| Nurses and/or midwives are not valued by society |  |  |  |  |  |
| My professional behaviour affects my image |  |  |  |  |  |
| Showing compassion and care is important |  |  |  |  |  |
| It is important for nurses and/or midwives to have good  customer service skills |  |  |  |  |  |
| Marketing nursing and/or midwifery to the public is  important |  |  |  |  |  |
| Humanity is an essential component of my role |  |  |  |  |  |
| The public has a positive image of nursing and/or midwifery |  |  |  |  |  |
| Nursing and/or midwifery have varied career paths |  |  |  |  |  |
| Nursing and/or midwifery is challenging and rewarding |  |  |  |  |  |

4.1 For members of the public who value and appreciate the role of nurses and/or midwives, what is the aspect they value most? *(List top three attributes)*

**5. Perceived Role of nurses and/or midwives**

5.1 In your opinion, what is the role of the nurse and/or midwife in your context? *(list top three)*

5.2 What *top three words* come to mind if you describe the *attitude* of doctors towards nurses and/or midwives?

5.3 What *top three words* come to mind if you describe what doctors perceive as the *role* of nurses and/or midwives?

5.4 What *top three words* come to mind if you describe the *attitude* of members of the public towards nurses and/or midwives?

5.5 In your opinion, how do members of the public perceive the *role* of nurses and/or midwives?

5.6 Do you think how the public perceives nursing and/or midwifery determines how nurses and/or midwives conduct their work?

o Yes/ No

If yes, explain *(word limit)*

**6. Changing the perception of nursing and midwifery profession**

a) What can nurses and/or midwives do to improve the service users/ public’s perception of their work?

List *(top three)*

Please add any further comments you may have:

**About You:**

What is your age?

What is your gender? Male 🞏 Female 🞏

**What is your professional group/designation?** Enrolled Nurse 🞏 Enrolled Midwife 🞏

Registered nurse 🞏

Registered midwife 🞏

Registered community health nurse 🞏

**What is your highest qualification?** Certificate 🞏 Diploma 🞏

Degree 🞏

Masters 🞏

PhD 🞏

How many years have you practiced as a nurse?

In your current job, do you offer direct patient care? Yes 🞏 No 🞏

Thank you for completing this form
